# Human antibody responses against non-covalently cell wall-bound *Staphylococcus aureus* proteins

**DOI:** 10.1038/s41598-018-21724-z

**Published:** 2018-02-19

**Authors:** Francisco Romero Pastrana, Jolanda Neef, Dennis G. A. M. Koedijk, Douwe de Graaf, José Duipmans, Marcel F. Jonkman, Susanne Engelmann, Jan Maarten van Dijl, Girbe Buist

**Affiliations:** 1 Department of Medical Microbiology, University of Groningen, University Medical Center Groningen, Hanzeplein 1, P.O. Box 30001, 9700 RB Groningen, The Netherlands; 2 Department of Dermatology, University of Groningen, University Medical Center Groningen, Hanzeplein 1, P.O. Box 30001, 9700 RB Groningen, The Netherlands; 3 Institute of Microbiology, Technical University Braunschweig, Inhoffenstrasse 7, D-38124 Braunschweig, Germany; 4 Helmholtz Institute for Infection Research, Microbial Proteomics, Inhoffenstrasse 7, D-38124 Braunschweig, Germany

## Abstract

Human antibody responses to pathogens, like *Staphylococcus aureus*, are important indicators for *in vivo* expression and immunogenicity of particular bacterial components. Accordingly, comparing the antibody responses to *S. aureus* components may serve to predict their potential applicability as antigens for vaccination. The present study was aimed at assessing immunoglobulin G (IgG) responses elicited by non-covalently cell surface-bound proteins of *S. aureus*, which thus far received relatively little attention. To this end, we applied plasma samples from patients with the genetic blistering disease epidermolysis bullosa (EB) and healthy *S. aureus* carriers. Of note, wounds of EB patients are highly colonized with *S. aureus* and accordingly these patients are more seriously exposed to staphylococcal antigens than healthy individuals. Ten non-covalently cell surface-bound proteins of *S. aureus*, namely Atl, Eap, Efb, EMP, IsaA, LukG, LukH, SA0710, Sle1 and SsaA2, were selected by bioinformatics and biochemical approaches. These antigens were recombinantly expressed, purified and tested for specific IgG responses using human plasma. We show that high exposure of EB patients to *S. aureus* is mirrored by elevated IgG levels against all tested non-covalently cell wall-bound staphylococcal antigens. This implies that these *S. aureus* cell surface proteins are prime targets for the human immune system.

## Introduction

*Staphylococcus aureus* is a Gram-positive bacterial pathogen that colonizes about one third of the healthy human population^1^. The pathology caused by *S. aureus* may range from mild skin infections to life-threatening bacteremia. Current treatment of *S. aureus* infections relies on antibiotics, but the emergence of highly drug-resistant lineages^2^ has reignited the interest in alternative treatment options, including passive and active immunization^3–7^.

Surface-exposed and secreted proteins of *S. aureus* play pivotal roles in the colonization and subversion of the human host^8^. Accordingly, such proteins have been considered as possible antigens for vaccination against *S. aureus* infections^9,10^. However, the previous efforts to develop a vaccine against *S. aureus* have met with little success, as exemplified by trials based on capsular polysaccharides or important virulence factors, such as fibronectin binding protein (FnBP), collagen binding protein (CnBP), or clumping factor A (ClfA)^5,9,11^. Most likely, this relates to the broad spectrum of virulence and immune evasion factors that *S. aureus* employs to thrive and survive in the human host. Therefore, it has been suggested that potentially successful vaccines need to address multiple staphylococcal virulence factors and defense mechanisms^5^.

The *S. aureus* genome encodes about 2700 proteins, from which about 120 have been observed more than once in the extracellular and cell surface proteomes^10,12,13^. Since the pioneering experiments published by Etz *et al*. and Vytvytska *et al*. in 2002^14,15^, diverse immunological and proteomics-based studies have addressed the antibody responses against *S. aureus*^9,16^. In these studies the antigenicity of non-covalently cell wall-associated proteins received relatively little attention. These non-covalently cell wall-associated proteins include proteins with specific cell wall-binding domains, ‘secretable expanded repertoire adhesive molecules’ (SERAMs) and typical cytoplasmic proteins that are bound to the cell wall through as yet undefined mechanisms^17^. Members of this group are tissue adhesins, toxins and immune evasion factors. Since the functions of these proteins could be neutralized by effective antibody responses, they might be attractive targets for vaccination, provided that they are immunogenic. Recent reports have shown high immune responses against some members of this group, including IsaA, Efb and Atl^18,19^. Yet, in animal models it was shown that antibodies against these antigens provide only limited protection against challenges with *S. aureus*^6,20^.

Healthy immune-competent individuals display differing antibody responses to a vast array of *S. aureus* antigens, possibly reflecting their history of close encounters with multiple different *S. aureus* lineages^21,22^. Anti-staphylococcal antibody levels can increase strongly during bacteremia^21,23^, and it has been proposed that continuous exposure to different staphylococcal antigens might improve the effectiveness of the immune response^22^. Patients with the genetic blistering disease epidermolysis bullosa (EB) develop wounds that are highly susceptible to *S. aureus* colonization. Especially the chronic wounds of EB patients are heavily colonized with *S. aureus* and usually contain several different types of this pathogen^24–27^. This severe colonization of the wounds of EB patients is reflected in the very high anti-staphylococcal immunoglobulin G (IgG) levels in their plasma and blister fluid compared to the respective IgG levels in the plasma of healthy age-matched volunteers^19,28^. Importantly, also IgG4 responses against various *S. aureus* antigens were elevated in the plasma of EB patients, which is consistent with their long-term and/or repeated exposure to these antigens^28^. Remarkably, *S. aureus* bacteremia is infrequently observed in adult EB patients, suggesting that their anti-staphylococcal immune responses may be protective against invasive *S. aureus* infections^19^. Of note, in previous studies on the antibody responses of EB patients to *S. aureus* antigens, the non-covalently cell wall-bound proteins were underrepresented^19,28^.

The aim of the present exploratory study was to assess to what extent non-covalently cell wall-bound proteins of *S. aureus* are immunogenic and whether the respective IgG titers are elevated in plasma samples from EB patients. Based on a bioinformatics inventory and on data from our previous proteomics analyses of the *S. aureus* surfacome^29^, 10 non-covalently cell wall-bound proteins of *S. aureus* were selected, produced in *Lactococcus lactis*, and purified. The purified proteins were used to assess specific IgG levels in plasma samples from EB patients and healthy volunteers.

## Materials and Methods

### Bacterial strains, plasmids and growth conditions

Strains and plasmids used in this study are listed in Table 1. *L. lactis* strains were grown at 30 °C in M17 broth (Oxoid Limited, Hampshire, UK) supplemented with 0.5% glucose (wt/vol) (GM17). When necessary the medium was supplemented with chloramphenicol (5 µg/ml) or erythromycin (5 µg/ml) for plasmid selection. *S. aureus* strains were grown at 37 °C, 250 rpm in Tryptone Soy Broth (TSB; Oxoid). *E. coli* strain MC1061 was grown at 37 °C, 250 rpm in Lysogeny broth (LB; Becton Dickinson, Breda, The Netherlands). When necessary, the medium was supplemented with ampicillin (100 µg/ml) for plasmid selection.

**Table 1:** Strains and plasmids used in this study.

Strain or plasmid	Relevant phenotype(s) or genotype(s)	Source or reference
**Strains**		
*L. lactis* PA1001	MG1363 *pepN*::*nisRK*, allows nisin-inducible expression, Δ*acmA* Δ*htrA*	^46^
*E. coli* MC1061	*araD*139 Δ(araA-leu)7697 Δ*lacX*74 *galK*16 *galE*15(GalS) λ- e14- *mcrA*0 *relA*1 *rpsL*150(*strR*) *spoT*1 *mcrB*1 *hsdR*2	(Novagen, Madison, Wis)
*S. aureus* NCTC 8325	Propagating strain for typing phage 47	^64^
*S. aureus* Newman	NCTC 8178 clinical isolate	^65^
*S. aureus* Newman Δ*spa* Δ*sbi*	*S. aureus* Newman *spa sbi* mutant	^31^
**Plasmids**		
pRE-USPnlic	*amp*^*R*^; *cam*^*R*^; pRExLIC fused with *E. coli* pBR322 replicon	^33^
pERL	*ery*^*R*^; pERL fused with pSH71 replicon	^33^
pNG4210	*cam*^*R*^ pNG400 derivative, containing *Bam*HI/*Eco*RI-*Xba*I/*Not*I cloning sites, *his*_6_ followed by a Stop codon	^35^
pNZ:LIC:*efb*	Fusion of pRE-USPnlic with pERL containing *efb* (SAOUHSC_01114, aa 30–165)	This study
pNZ:LIC:*eap*	Fusion of pRE-USPnlic with pERL containing *eap* (SAOUHSC_02161, aa 31–584)	This study
pNZ:LIC:*sle1*	Fusion of pRE-USPnlic with pERL containing *sle1* (SAOUHSC_00427, aa 26–334)	This study
pNZ:LIC:*sa0710*	Fusion of pRE-USPnlic with pERL containing *sa0710* (SAOUHSC_00773, aa 25–279)	This study
pNZ:LIC:*atl1*	Fusion of pRE-USPnlic with pERL containing *atl1* (SAOUHSC_00994, aa 199–775)	This study
pNZ:LIC:*atl2*	Fusion of pRE-USPnlic with pERL containing *atl2* (SAOUHSC_00994, aa 776–1256)	This study
pNG4210:*lukG*	pNG4210 containing *lukG* with C-terminal *his*_6_ (SAOUHSC_02241, aa 30–338)	This study
pNG4210:*lukH*	pNG4210 containing *lukH* with C-terminal *his*_6_ (SAOUHSC_02243, aa 33–351)	This study
pNG4210:*ssaA2*	pNG4210 containing *ssaA2* with C-terminal *his*_6_ (SAOUHSC_02571, aa 28–267)	This study
pNG4210:*emp*	pNG4210 containing *emp* with C-terminal *his*_6_ (SAOUHSC_00816, aa 27–340)	This study
pNG4210:*ftsL*	pNG4210 containing *ftsL* with C-terminal *his*_6_ (USA300HOU_1120, aa 66–133)	^35^

*Aa*, amino acid residue; *amp*^*R*^, ampicillin resistance gene; *cam*^*R*^, chloramphenicol resistance gene; e*ry*^*R*^, erythromycin resistance gene; P_*nisA*_, nisin-inducible promoter; *usp45*_ss_, signal sequence of *usp45*.

### Isolation of *S. aureus* cell wall fragments

Cell wall fragments (CWFs) from *S. aureus* were isolated as described previously^30^. In short, *S. aureus* Newman cells were collected by centrifugation, glass-beads were added (0.1 µm beads, Biospec Products, Bartlesville, USA), and cells were disrupted for 2 min in a Precellys 24 homogenizer (Bertin Technologies, Saint Quentin en Yvelines Cedex, France). The resulting CWFs were collected by centrifugation and boiled at 96 °C for 10 min in 4% sodium dodecyl sulphate. This step was repeated twice. CWFs were subsequently washed six times with Phosphate Buffered Saline (PBS) and stored at −20 °C until further use.

### Identification of non-covalently cell surface-bound proteins

**Figure 1:**
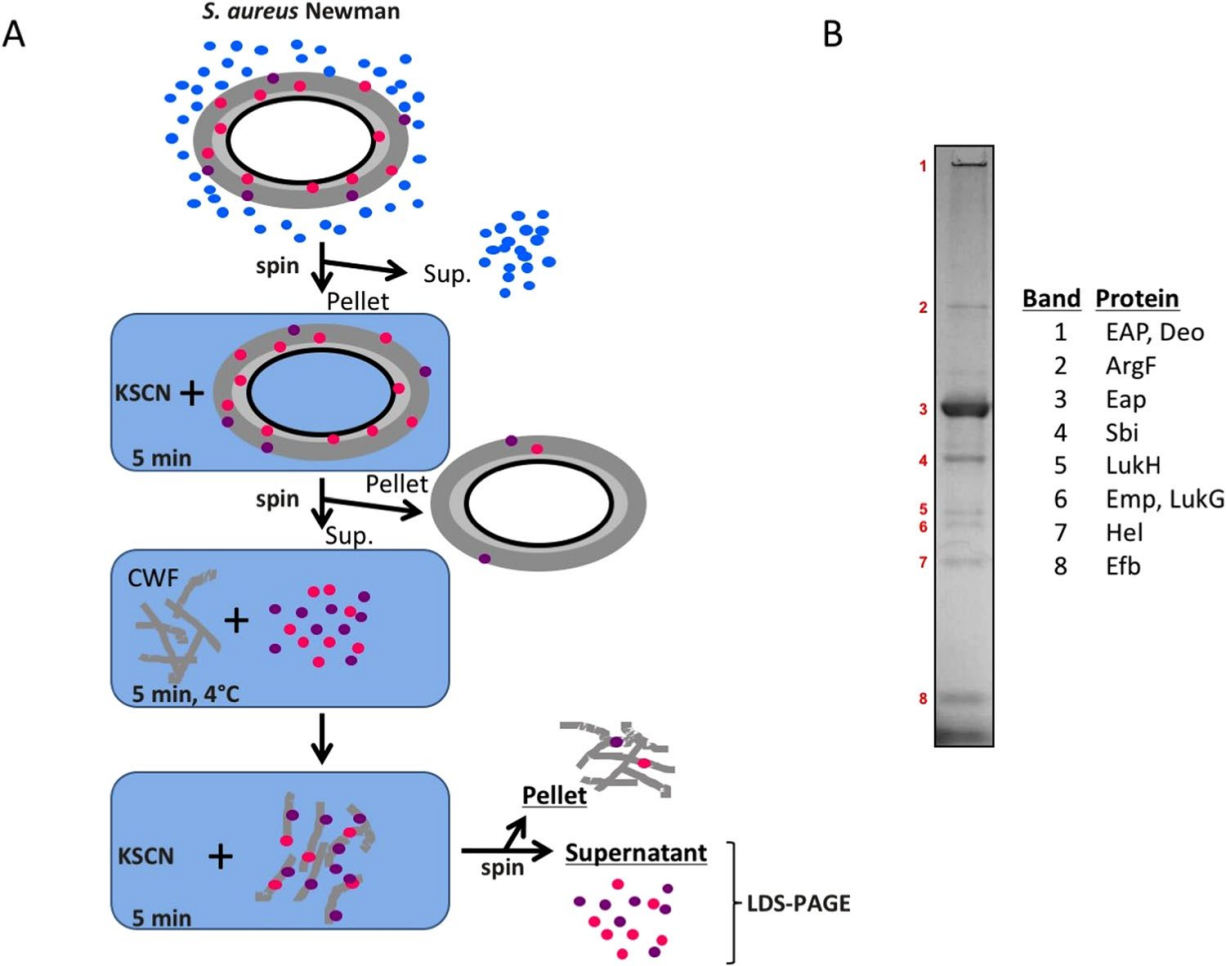
Identification of non-covalently cell wall-bound *S. aureus* proteins. (**A**) Schematic representation of the experimental set-up for identification of non-covalently cell wall-bound proteins. *S. aureus* cells were first separated from the growth medium by centrifugation (spin). Pelleted *S. aureus* cells were treated with KSCN to release the non-covalently cell wall-bound proteins. KSCN-extracted proteins were re-bound to cell wall fragments (CWF) and, subsequently, released again by KSCN incubation. Upon centrifugation, the resulting pellet and supernatant fractions were analyzed by LDS-PAGE (**B**). Upon Simply Blue safe staining of the gel, protein bands were excised and identified by MS as indicated.

Non-covalently cell wall-bound proteins of *S. aureus* were identified by using the amino acid sequences of known domains for non-covalent cell wall-binding (i.e. PROSITE PS51780, PS51782, PS51781, PS51109) in BLAST searches against the sequenced *S. aureus* Newman strain. The actual identification of expressed non-covalently cell wall-bound proteins was accomplished as schematically represented in Fig. 1A. Upon overnight culturing in TSB, *S. aureus* Newman cells were incubated with 2 M potassium thiocyanate (KSCN) for 5 min leading to the release of non-covalently cell wall-bound proteins. Liberated non-covalently cell wall-bound proteins in the soluble fraction were either TCA-precipitated or dialyzed against PBS using a 3,500 Molecular weight cut-off (MWCO) membrane (Spectrum laboratories Inc. USA) as described before^31^. Secreted proteins present in *S. aureus* Newman growth medium fractions were also collected, either by TCA precipitation or dialysis of the spent growth medium against PBS. Dialyzed non-covalently cell wall-bound proteins were added to prepared *S. aureus* CWFs and incubated for 5 min at 4 °C unless stated otherwise. CWFs containing non-covalently bound proteins were washed with PBS and non-covalently cell wall-bound proteins were released into the supernatant fraction by incubation with 2 M KSCN as described above. Released proteins were collected either by TCA precipitation or dialyzed overnight against demineralized water. Released proteins and cell pellets were separated by lithium dodecyl sulphate (LDS) - PAGE and the respective gels were stained with SimplyBlue SafeStain (Life Technologies, Grand Island, NY. USA). Protein bands were cut from the gels (Fig. 1B) and identified by Mass Spectrometry (MS) as described previously^32^.

### Expression of non-covalently cell wall-bound proteins in *L. lactis*

PCR was performed with the *Pwo* DNA polymerase (Roche Diagnostics, Woerden, The Netherlands), using chromosomal DNA of *S. aureus* NCTC 8325 as template. Primers listed in Table 2 were designed to amplify gene sequences without the region coding for the natural secretion signals and with 5′ end extensions for ligase-independent cloning (LIC)^33^. Briefly, *Swa*I-digested pRE-USP plasmid and PCR fragments were treated with T4 DNA polymerase (20 °C, 20 min; 75 °C, 20 min; Roche Diagnostics) before incubation for 5 min at room temperature (3:1 vector:insert). Z-Competent *E. coli* MC1061 cells (Zymo Research, Orange, CA, USA) were transformed with the plasmid:vector mixtures. Correct plasmids were confirmed by DNA sequencing (Eurofins DNA, Germany). For cloning in *L. lactis*, Vector Backbone Exchange was performed by mixing ~300 ng of pERL vector with ~300 ng of the pRE-USP harboring the gene of interest, both digested with *Sfi*I (New England Biolabs, Ipswich, UK). Ligation was performed using T4 DNA Ligase (New England Biolabs) and the resulting vector was introduced into electrocompetent *L. lactis* PA1001^34^. For insertion of genes into plasmid pNG4210, primers (Table 2) were designed to amplify the respective sequences without the region coding for the natural secretion signals and with 5′ end extensions encoding *Bam*HI (forward) or *Not*I (reverse) restriction sites. Briefly, digested PCR products and linearized plasmid were separated by agarose gel electrophoresis, and selected DNA fragments were gel-extracted and purified. Ligation of digested plasmid and PCR fragments was performed using T4 DNA ligase and the resulting plasmid was introduced into electrocompetent *L. lactis* PA1001 as described before^35^. For the expression of cloned genes, *L. lactis* cultures were induced in the exponential phase of growth (0.5 O.D. at 600 nm) by the addition of nisin (final concentration 3 ng/ml, Sigma-Aldrich, St. Luis, MO). After 4 h or overnight culturing, the cells were separated from the growth medium by centrifugation. Proteins in the nisin-induced growth medium fractions were precipitated with TCA (10% W/V) and resuspended in LDS gel loading buffer (Life Technologies). Cells in LDS sample buffer were disrupted with 0.1 µm glass beads in a Precellys 24 homogenizer. Both cellular and growth medium fractions were analyzed by LDS-PAGE (Life Technologies) and proteins were either visualized using protein staining with the SimplyBlue SafeStain (Life Technologies), or by blotting onto nitrocellulose membranes (Protan nitrocellulose transfer paper, Whatman, Germany) and subsequent immunodetection. For immunodetection, mouse anti-his tag primary antibodies (Life Technologies) and fluorescently labeled secondary antibodies (goat anti-mouse IRDye 800 CW, LI-COR Biosciences, Lincoln, NE. USA) were used. Antibody binding was visualized with an Odyssey infrared imaging system (LI-COR Biosciences).

**Table 2:** Primer sequences used in this study.

Primers	5′ → 3′ nucleotide sequence	
*efb*.fw	ATGGTGAGAATTTATATTTTCAAGGTAGCGAAGGATACGGTCCAAG	
*efb*.rev	TGGGAGGGTGGGATTTTCATTATTTAACTAATCCTTGTTTTAATACATTATC	
*eap*.fw	ATGGTGAGAATTTATATTTTCAAGGTGCAGCTAAGCCATTAGATAAATC	
*eap*.rev	TGGGAGGGTGGGATTTTCATTATTTATTTTTTTTTGATTTAGTGTATTG	
*sle1*.fw	ATGGTGAGAATTTATATTTTCAAGGTGCTACAACTCACACAGTAAAAC	
*sle1*.rev	TGGGAGGGTGGGATTTTCATTAGTGAATATATCTATAATTATTTACTTGGT	
*sa0710*.fw	ATGGTGAGAATTTATATTTTCAAGGTCAACAACATGGCACACAAG	
*sa0710*.rev	TGGGAGGGTGGGATTTTCATTAGTGGATGTAATTATATTTTCCTG	
*atl(1)*.fw	ATGGTGAGAATTTATATTTTCAAGGTGCTTCAGCACAACCAAG	
*atl(1)*.rev	TGGGAGGGTGGGATTTTCATTATTTTACAGCTGTTTTTGG	
*atl(2)*.fw	ATGGTGAGAATTTATATTTTCAAGGTGCTTATACTGTTACTAAACCACAAAC	
*atl(2)*.rev	TGGGAGGGTGGGATTTTCATTATTTATATTGTGGGATGTCG	
*lukG.fw*	ATATGGATCCAAGATTAATTCTGAAATCAAACAAGTTTCTG	*Bam*HI
*lukG.rev*	ATATGCGGCCGCTTTCTTTTCATTATCATTAAGTACTTTTAC	*Not*I
*lukH.fw*	ATATGGATCCGACTCTCAAGACCAAAATAAGAAAG	*Bam*HI
*lukH.rev*	ATATGCGGCCGCTCCTTCTTTATAAGGTTTATTGTCATC	*Not*I
*ssaA2.fw*	ATATGGATCCTCTGAGCAAGATAACTACGGTTATAATCC	*Bam*HI
*ssaA2.rev*	ATATGCGGCCGCGTGAATGAAGTTATAACCAGCAGCTTGG	*Not*I
*emp.fw*	ATATGGATCCTCAGTGACAGAGAGTGTTGAC	*Bam*HI
*emp.rev*	ATATGCGGCCGCTACTCGTGGTGCTGGTAAG	*Not*I

Underlined are the LIC cloning sequences/restriction sites.

### Protein purification and activity measurements

When expressed proteins remained cell-associated, they were liberated from the cells either with 2 M KSCN or 6 M urea, as required. Next, the protein-containing soluble fractions were separated from the cell fraction by centrifugation. Subsequently, his-tagged proteins were purified from the respective supernatant fractions using the HisLink Protein Purification resin (Promega Corporation, Madison, WI. USA), in the absence or presence of either 2 M KSCN or 6 M urea. The HisLink binding and washing buffer was composed of 0.1 M HEPES 7.5 pH, 0.5 M NaCl and 10 mM imidazole. The elution buffers were essentially the same, but contained 200 mM or 400 mM imidazole. The IsaA and FtsL proteins were purified as described previously^35,36^.

### Rebinding of isolated proteins to *S. aureus* cells

Overnight growth cultures of *S. aureus* Newman Δ*spa*Δ*sbi* were resuspended to 1 optical density measured at 600 nm in 800 µl of PBS (pH 7) or 50 mM sodium acetate (pH 5) and incubated with 1–3 µg of histidine-tagged fusion proteins for 10 min. After incubation, cell pellets and supernatants were processed, and localization of tagged proteins was assessed by LDS-PAGE and Western blotting as described above.

### Enzyme-linked immunosorbent assay (ELISA)

Plasma samples were previously donated by patients with EB from the Dutch Epidermolysis Bullosa Registry (DEBR), and by healthy volunteers from the Netherlands. The EB patients included six patients with junctional EB (EB01, EB02, EB09, EB15, EB53, EB60), one patient with EB simplex (EB11), and one patient with dystrophic EB (EB51)^19^. ELISA plates were coated overnight at 4 °C with histidine-tagged fusion proteins (100ng/well) diluted in carbonate coating buffer (50 mM sodium carbonate, pH 9.6)^6^. Subsequently, the plates were blocked with PBS containing 5% skim milk. Patient and healthy control plasma samples were processed as previously described^19^. Serial dilutions of plasma (1000- to 2,000,000-fold) were prepared in PBS-Tween 20/5% skim milk. Specific anti-human IgG secondary antibodies coupled to horseradish peroxidase (dilution 1:8,000; Southern Biotechnology, Birmingham, AL) were used according to the manufacturer’s recommendations. Horseradish peroxidase activity was quantified by measuring the hydrolysis of the substrate (O-Phenylenediamine, Sigma-Aldrich) at OD_492_ in a plate reader (Biotek Powerwave XS2, USA). The raw ELISA data are provided in Supplementary Information Table 1. Titers were calculated in arbitrary units (AU) through extrapolation using linear regression for data points from known dilutions giving OD_492_ values between 0.1 and 1.0. All calculated R^2^ linear regression values (Pearson product moment correlation coefficient) were above 0.98. IgG titers in plasma samples of EB patients were averaged and normalized by adjusting the averaged IgG titers in the control plasma samples of healthy volunteers to a single arbitrary level (AU = 10) and adjusting accordingly the averages for the EB patient samples. In brief, obtained EB and control averages were multiplied by a numeric factor that resulted in the average of all controls equal to 10 AU. After plotting normalized values, the differences in the average IgG levels measured for plasma samples from EB patients and healthy control individuals were compared for the different analyzed proteins. Statistical significance of the ELISA data was tested with a Mann-Whitney *U* test for two independent samples (Supplementary Information Table 1).

### Ethics statements

Blood donations from EB patients were collected with approval of the medical ethics committee of the University Medical Center Groningen (approval no. NL27471,042,09). The Independent Ethics Committee of the Foundation ‘Evaluation of Ethics in Biomedical Research’ (Assen, the Netherlands), approved the protocol for blood donations from healthy volunteers, which is registered by QPS Groningen (code 04132-CS011). Blood donations were obtained after written informed consent from all eight EB patients and the six healthy volunteers included in this study, and adhering to the Helsinki Guidelines^19^.

## Results and Discussion

### Selection of non-covalently cell wall-bound proteins

To pinpoint a panel of non-covalently cell wall-bound proteins of *S. aureus*, we performed an extensive bioinformatics analysis using the genome sequence of *S. aureus* strain Newman and, in addition, we surveyed the results of our previous analysis of the cell surface proteome of this *S. aureus* strain^29^. The results are summarized in Table 3. Specifically, the bioinformatics approach identified several SERAMS, in particular the extracellular adhesive protein Eap, the extracellular matrix protein Emp, the extracellular fibrinogen-binding protein Efb, and coagulase^37^. Furthermore, we retrieved all known *S. aureus* Newman proteins containing the conserved cell wall-binding domains LysM (PROSITE: PS51782)^38,39^, GW (PROSITE: PS51780)^40^, SH3B (PROSITE: PS51781)^39,41^ and G5 (PROSITE: PS51109)^42^. Except for the amidase from phage phiNM2 and the transmembrane protein EbpS, all other identified proteins carried a predicted signal peptide for export from the cytoplasm (indicated as *S* in Table 3). Of note, Eap, Atl, IsaA, SsaA2, Sle1, LukG and LukH have also previously been identified as non-covalently cell wall-bound proteins^16,29,43,44^. Further, Sle1 was shown to be localized in the vicinity of the *S. aureus* cross-wall^43,45^, while Atl was found to be preferably bound to the septal region^44^.

**Table 3:** Identified non covalently cell surface-bound proteins.

Uniprot ID	Gene names	AA	*S* ^e^	Protein name
**Proteins with known conserved non covalently cell wall binding domains**				
**LysM (PS51782)** ^a^				
A6QH29	NWMN_1389 *ebpS*	486		Elastin-binding protein EbpS
A0A0H3K686	NWMN_0055 *spa*	520	*S*	Immunoglobulin G binding protein A
A0A0H3K6S5	NWMN_0724 SA0710^c^	279	*S*	Uncharacterized protein
A0A0H3K7F5	NWMN_0634	265	*S*	Secretory antigen SsaA-like protein
A0A0H3KAZ4	NWMN_0429 *sle1*^d^	334	*S*	N-acetylmuramoyl-L-alanine amidase AAA
A0A0H3KG37	NWMN_1157 *lytN*	383	*S*	Cell-wall hydrolase LytN
**GW (PS51780)** ^**a**^				
A0A0H3K7X7	NWMN_0922 *atl*	1256	*S*	Bifunctional autolysin
**SH3B (PS51781)** ^**a**^				
A0A0H3K875	NWMN_1039	481		Phage amidase [Bacteriophage phiNM2]
A0A0H3K8J7	NWMN_1534	291	*S*	Probable cell wall amidase LytH
**G5** **(PS51109)** ^a^				
A0A0H3KAI3	NWMN_2392	1501	*S*	Uncharacterized protein
**Identified by surfacome profiling**				
A0A0H3K7X7	NWMN_0922 *atl*	1256	*S*	Bifunctional autolysin
A6QIG7	NWMN_1877 *chp*	149	*S*	Chemotaxis inhibitory protein precursor
A0A0H3KA75	NWMN_0166 *coa*	636	*S*	Coagulase
A6QF98	NWMN_0758 *emp*^d^	340	*S*	Extracellular matrix protein-binding protein emp precursor
A0A0H3KF27	NWMN_2399 *fnbA*	741	*S*	Fibronectin binding protein A
A0A0H3K6R0	NWMN_0687	646	*S*	Lipoteichoic acid synthase
A6QK59	NWMN_2469 *isaA*	233	*S*	Probable transglycosylase IsaA precursor
A6QJQ7	NWMN_2317 *sbi*	436	*S*	Immunoglobulin-binding protein sbi precursor
A0A0H3KCA1	NWMN_1066	109	*S*	Uncharacterized protein Ehp
A0A0H3K6X4	NWMN_0362	203	*S*	Uncharacterized protein
A0A0H3KET4	NWMN_0585	168	*S*	Uncharacterized protein
A0A0H3K7N7	NWMN_0757	508	*S*	Secreted von Willebrand factor-binding protein
A0A0H3KEG7	NWMN_2199 *ssaA2*^d^	267	*S*	Secretory antigen SsaA
**LDS-PAGE: Gel band MS**				
1^b^ A6QIG2	NWMN_1872 *eap*^d^	584	*S*	65 kDa membrane protein precursor
A6QDC3	NWMN_0083 *deoB*	392		Phosphopentomutase
2 A6QG68	NWMN_1078 *argF*	333		Ornithine carbamoyltransferase
3 A6QIG2	NWMN_1872 *eap*^d^	584	*S*	65 kDa membrane protein precursor
4 A6QJQ7	NWMN_2317 *sbi*	436	*S*	Immunoglobulin-binding protein Sbi precursor
5 A0A0H3KHT5	NWMN_1928 *lukH*^d^	351	*S*	Leukocidin/hemolysin toxin family S subunit
6A6QF98	NWMN_0758 *emp*^d^	340	*S*	Extracellular matrix protein-binding protein Emp precursor
A0A0H3K9N1	NWMN_1927 *lukG*^d^	338	*S*	Leukocidin/hemolysin toxin family F subunit
7A0A0H3K5Z1	NWMN_0249 *hel*^d^	296	*S*	5′-nucleotidase, lipoprotein e(P4) family protein
A0A0H3KIY0	NWMN_2444	85		Uncharacterized protein
8A6QG59	NWMN_1069 *efb*^d^	165	*S*	Fibrinogen-binding protein precursor

^a^PROSITE ID of Motif; ^b^Extracted band number from LDS-PAGE; ^c^Gene locus of NWMN_0724 homolog in *S. aureus* N315, used instead of gene name; ^d^Gene name of homologous protein if none was found in the Uniprot record; *S*^e^ Secretion signal predicted by SignalP and/or according to Uniprot.

To detect the actually produced non-covalently cell wall-bound proteins of *S. aureus* Newman and to ensure that the identified proteins do indeed behave as non-covalently cell wall-bound proteins, we extracted these proteins with the chaotrope KSCN from the staphylococcal cells, reattached them to isolated *S. aureus* CWFs, and re-extracted the proteins with KSCN (schematically represented in Fig. 1A). The proteins thus obtained were separated by LDS-PAGE. Eight dominant bands were detected, excised from the gel and identified by MS (Fig. 1B). The identifiers and characteristics of the identified proteins are summarized in Table 3. In this respect, it is noteworthy that our unpublished proteomics data indicate that only about 16% of the KSCN-extractable proteins are specifically cell wall-bound proteins.

**Figure 2:**
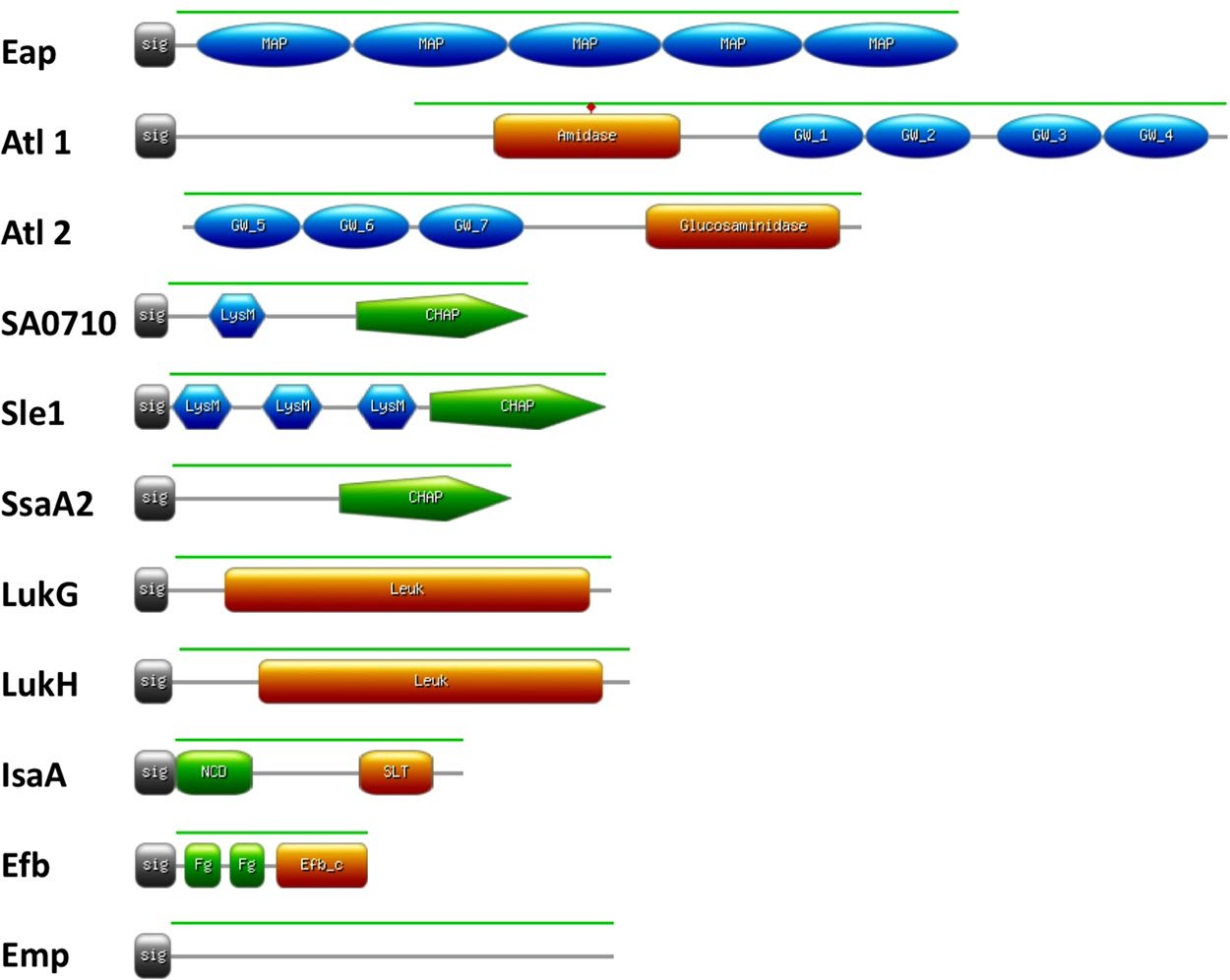
Motif composition of non-covalently cell wall-bound proteins of *S. aureus*. The proteins shown are: the extracellular adherence protein Eap (SAOUHSC_02161); the bifunctional autolysin Atl (SAOUHSC_00994), of which the Atl1 (N-acetylmuramoyl-L-alanine amidase) and Atl2 (Endo-beta-N-acetylglucosaminidase) domains were separately expressed; the CHAP and LysM domain-containing protein SA0710 (SAOUHSC_00773); the N-acetylmuramoyl-L-alanine amidase Sle1 (SAOUHSC_00427); the staphylococcal secretory antigen SsaA2 (SAOUHSC_02571); the gamma-hemolysin subunit B LukG (SAOUHSC_02241); the leukocidin LukH (SAOUHSC_02243); the probable transglycosylase IsaA (SA2356); the fibrinogen-binding protein Efb (SAOUHSC_01114); and the extracellular matrix protein-binding protein Emp (SAOUHSC_00816). Sig, signal peptide; MAP, MAP repeat profile (PROSITE: PS51223); amidase, N-acetylmuramoyl-L-alanine amidase (Pfam: PF01510); glucosaminidase, endo-beta-N-acetylglucosaminidase (Pfam: PF01832); GW, GW domain profile (PROSITE: PS51780); LysM, LysM domain profile (PROSITE:PS51782); CHAP, CHAP domain profile (PROSITE: PS50911); Leuk, Leukocidin/Hemolysin toxin family (Pfam: PF07968); NCD, N-terminal conserved domain; SLT, Transglycosylase SLT domain (Pfam: PF01464); Efb_c, extracellular fibrinogen binding protein C terminal (Pfam: PF12199); Fg, fibrinogen-binding motifs^63^. The green line represents amino acid residues selected for cloning and expression.

For our further studies on human antibody responses against non-covalently cell wall-bound proteins of *S. aureus*, we made a selection of 10 representative proteins. The inclusion criteria for these 10 proteins were identification by bioinformatics and/or biochemical analysis. Exclusion criteria were a lack of identification in previous biochemical or proteomics analyses^29^, the absence of a predicted signal peptide, the presence of an LPxTG motif for covalent cell wall binding, and known IgG-binding properties that would interfere with our further analyses. The domain structure of the selected proteins, highlighting domains potentially involved in cell wall binding, is represented in Fig. 2. It should be noted that Atl is synthesized in a pre-pro-form which, upon export, is cleaved into two moieties with an amidase domain (here termed Atl1) and a glucosamidase domain (here termed Atl2). Accordingly, the Atl2 moiety of Atl does not have its own signal peptide for export from the cytoplasm.

### Cloning, production and isolation of non-covalently cell wall-bound proteins in *L. lactis*

**Figure 3:**
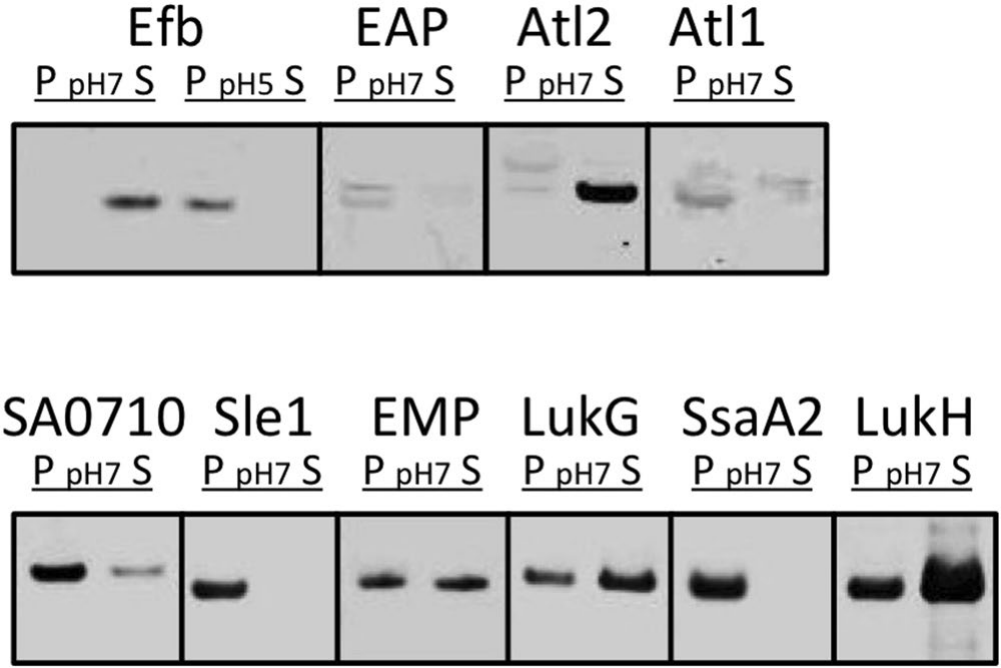
Binding of purified non-covalently cell wall-bound proteins to *S. aureus* cells. Non-covalently cell wall-bound proteins were expressed in *L. lactis* and their binding to whole cells of *S. aureus* was assessed upon incubation at pH 7, or pH 5 in the case of Efb as indicated. Please note that the different groupings of blots (marked by boxes) were cropped per investigated protein from different parts of the same Western blot, or from different Western blots that were similarly processed and scanned. P, cell pellet fraction; S, supernatant fraction.

Genes for the selected non-covalently cell wall-bound proteins of *S. aureus* were cloned into nisin-inducible expression vectors and introduced into *L. lactis* strain PA1001 for expression. In the case of Atl, the Atl1 and Atl2 moieties were expressed separately, each being secreted with the lactococcal Usp45 signal peptide. Of note, the *L. lactis* PA1001 strain lacks the genes for the major extracellular protease HtrA and the autolysin AcmA, which minimizes proteolysis and cell lysis, respectively^36,46^. Next, expression of the cloned genes was induced with nisin and the subcellular localization of the respective *S. aureus* proteins was determined. To this end, cells were separated from the growth medium by centrifugation and the respective fractions were analyzed by LDS-PAGE and Western blotting using anti-His_6_ antibodies. As expected, all proteins were largely cell wall-bound (data not shown). By incubation of the cells with 2 M KSCN (Efb, Eap and Atl1) or 6 M urea (all seven other proteins), the expressed proteins were released, consistent with disruption of their non-covalent interactions with the cell wall. Upon centrifugation, the released proteins were purified from the resulting supernatant fractions using Ni-NTA agarose beads, and their potential to re-bind to cells of *S. aureus* Newman Δ*spa* Δ*sbi* was confirmed (Fig. 3; data not shown for IsaA). Notably, Efb did not re-bind to *S. aureus* cells under the standard assay conditions (pH 7), but its binding to the cells could be demonstrated at a lowered pH of 5 (Fig. 3). Altogether, these findings imply that the purified proteins have retained their cell wall-binding capabilities. Of note, the cell wall binding of SA0710 had not been experimentally verified so far, despite the fact that this protein has a LysM domain.

### Human IgG responses against non-covalently cell wall-bound proteins of *S. aureus*

**Figure 4:**
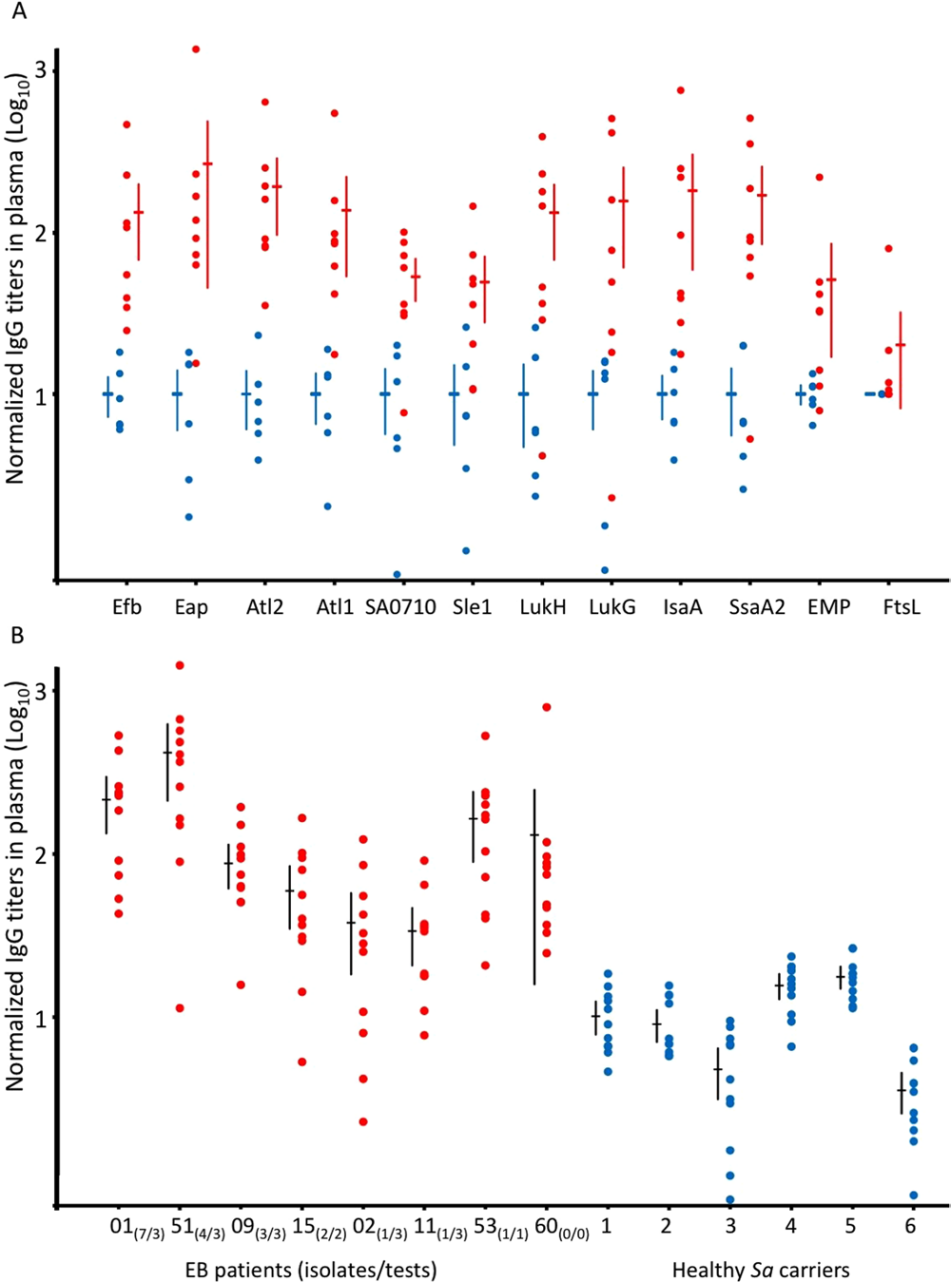
Binding of human IgGs to purified non-covalently cell wall-bound *S. aureus* proteins. Levels of IgGs specific for purified cell wall-bound *S. aureus* proteins were compared by ELISA using plasma samples of patients with EB (red bullets) or healthy *S. aureus* carriers (blue bullets). (**A**) Normalized IgG titers in different plasma samples plotted per purified *S. aureus* antigen. (**B**) Normalized IgG titers in different plasma samples plotted per EB patient and healthy carrier. In brief, obtained EB and control averages were multiplied by a numeric factor that resulted in the average of all controls equal to 10 AU. After plotting normalized values, the differences in the average IgG levels measured for plasma samples from EB patients and healthy control individuals were compared for the different analyzed proteins (isolates/tests). Per patient, the number of *S. aureus* types identified per number of sampling time points are indicated in parentheses^19,24,26^. *Sa*, *S. aureus*.

To assess whether EB patients and healthy volunteers mount immune responses against the selected non-covalently cell wall-bound proteins of *S. aureus*, we applied an ELISA approach. The membrane protein FtsL was included in this analysis as a control, because it is surface-exposed, but not bound to the cell wall^29^. As shown in Fig. 4A and Supplementary Information Table 1, all investigated human plasma samples contained IgGs against all investigated proteins. Importantly, the levels of IgGs against non-covalently cell wall-bound proteins in plasma samples from EB patients were, on average, about 10-fold higher than those from healthy carriers. In this respect, the largest differences were observed for Eap, Atl2 and IsaA, and the smallest for Sle1 and Emp. Only, the FtsL-specific IgG levels in plasma samples from EB patients and healthy volunteers did not differ significantly (Fig. 4A). In this respect, it should be noted that for many, but not all, *S. aureus* antigens elevated IgG levels were previously observed in plasma from EB patients^19,28^. For example, no significant differences in IgG levels of EB patients and healthy volunteers were previously observed for the ClfA, ClfB and IsdH proteins bound to the staphylococcal cell wall.

We further inspected the overall IgG responses to all non-covalently cell wall-bound proteins per plasma sample, excluding FtsL. As shown in Fig. 4B, the normalized average IgG levels against the eleven remaining *S. aureus* antigens were higher for the eight plasma samples from EB patients than for the six healthy volunteers. Further, the carriage of multiple *S. aureus* strains by EB patients correlated with higher normalized average IgG levels (Fig. 4B). Thus, the highest normalized average IgG levels were observed for patients EB01 and EB51 who, respectively, were previously shown to carry 7 and 4 different *S. aureus* types at 3 time points of sampling^19,28^.

During *S. aureus* colonization and invasion, immune-competent individuals may rapidly mount antibody responses to a large panel of antigens. The antibody response profiles of individuals usually reflects the history of previous encounters with *S. aureus*^22^. Accordingly, they may change after every new encounter^21^, which could explain the strong variations in the profiles observed for different individuals^28,47,48^. Importantly, this was previously shown to even be the case upon controlled nasal inoculation of healthy human volunteers with *S. aureus*^49^.

In previous studies, we have reported that patients with EB develop wounds that are highly susceptible to *S. aureus* colonization^19,24–26^. Accordingly, it was shown that these patients mounted significantly higher immune responses against *S. aureus* antigens than healthy carriers^19,28^. In these studies, compared to healthy control individuals, elevated IgG levels were observed in plasmas of EB patients for three out of eleven tested cell wall-associated antigens (i.e. Efb, IsaA and IsdA), and for seven out of seventeen tested secreted antigens. Minor differences were observed for IgG responses against secreted superantigens^19^. In the present exploratory study, we show that EB patients carry significantly increased IgG levels against all eleven tested antigens that are non-covalently bound to the *S. aureus* cell wall, including Efb and IsaA. This novel observation highlights the strong immunogenicity of these antigens compared to other staphylococcal antigens that are covalently bound to the cell wall or secreted into the host environment. When IgG levels were compared for EB patients carrying multiple *S. aureus* types versus EB patients carrying only one *S. aureus* type, significant differences were observed for 4 out of 10 tested antigens, again including Efb and IsaA^19^. This general difference upon colonization with multiple *S. aureus* types was also observed in the present study, supporting the view that patients exposed to different *S. aureus* types are challenged with more staphylococcal antigens than patients carrying only one *S. aureus* type. Another novel finding is that EB patients showed a much larger variation in IgG levels against *S. aureus* antigens than healthy carrier controls. For example, the level of IgG against LukG in EB patient 02 was lower than the respective IgG levels in four of the healthy control individuals, whereas EB patient 53 showed the highest of all presently recorded IgG levels against LukG. Additionally, the comparison of plasma samples from EB patients and healthy control individuals revealed no significantly different IgG levels for other surface-exposed proteins of *S. aureus*, like FtsL. Thus, while it has been previously shown that EB patients have in general higher levels of antibodies against *S. aureus* antigens, our present study shows that (i) not all surface-exposed *S. aureus* proteins will elicit a similar antibody response in each EB patient, and that (ii) different EB patients similarly exposed to *S. aureus* may generate highly diverse patterns of antibody responses against particular *S. aureus* antigens. This relates most likely to their *S. aureus* contact history, which was previously shown to be variable over time^19,24,26^.

The species *S. aureus* is known to display high genomic plasticity. Although, the genes for some virulence factors are (almost) invariantly (Efb, Eap, Emp, IsaA) or frequently (Atl, LukG, LukH, SsaA2, Sle1) present in *S. aureus* isolates, their amino acid sequence identity and expression levels can show substantial inter-strain variations^13,17,21,29,50–52^. This diversity could be a determinant for variations in the host immune responses to *S. aureus*. Our results with Eap and Efb, which present large inter-lineage amino acid sequence variation^51^, showed a highly variable immune response in IgG titers in EB patients compared to healthy carriers. Interestingly, previous studies on different (not EB-related) patient cohorts showed either lower levels of anti-Eap and Efb antibodies in patients^48,53,54^ or, on the contrary, higher levels in infected patients than in healthy *S. aureus* carriers^23,47,55,56^. Nonetheless, consistently higher IgG titers against Atl^14^ and IsaA^19^ in sera of *S. aureus-*infected or colonized patients have been previously reported, which is in agreement with our own results.

In the context of our present study, it is noteworthy that except for very severe cases, patients with EB appear to suffer infrequently from invasive staphylococcal disease, especially if one considers their high rates of colonization with *S. aureus*^28^. This has led to the proposal that the elevated levels of anti-staphylococcal IgGs could potentially be protective. Importantly, none of the patients who participated in this and our previous studies was treated for *S. aureus* bacteremia in the 5 years prior to donating the investigated plasma samples. Additional support for the idea that high anti-staphylococcal IgG levels in EB patients could be protective comes from studies with monoclonal antibodies against IsaA, showing protection against *S. aureus* infections in murine *S. aureus* infection models^6,7,57^. At present, it is not clear whether these findings for anti-IsaA antibodies can be extrapolated to other non-covalently cell wall-bound proteins. This idea is tempting in view of the present results, but it has to be noted that vaccination studies in murine models with an octa-valent vaccine, including Atl and IsaA, did not lead to protection against *S. aureus* challenges^18^. Yet, immune responses directed against other presently investigated antigens could be beneficial, not only by promoting opsonophagocytosis, but also by interfering with the biological activity of the different antigens as was recently shown for monoclonal antibodies against the staphylococcal complement inhibitor SCIN^58^. Clearly, potentially beneficial effects of using non-covalently cell surface-bound proteins as antigens for vaccination need to be thoroughly validated in immunization experiments. This is important, because strong immune responses against *S. aureus* antigens may not be (fully) protective. This is underscored by the observation that *S. aureus* carriers producing antibodies that neutralize superantigens can still develop sepsis, although they do have an improved prognosis^59^. Also, in animal experiments high antibody titers against *S. aureus* antigens, such as IsaA, are not necessarily protective against infections by this pathogen^18,60^. In this context, it is noteworthy that Hawkins *et al*. showed that natural exposure of humans to *S. aureus* elicited a strong antibody response against ClfA, but this response did not prevent the ClfA-mediated binding of *S. aureus* cells to fibrinogen, the natural ligand of ClfA^61^. In contrast, immunization with a ClfA-containing vaccine induced functional antibodies that prevented *S. aureus* from binding to fibrinogen. Another recent observation that encourages further research towards the development of an anti-staphylococcal vaccine was reported by Stentzel *et al*., who showed that immunoglobulin replacement therapy in STAT3 hyper-IgE syndrome patients with low *S. aureus*-specific serum IgG levels did not only increase the *S. aureus*-specific IgG levels, but also resulted in an attenuated clinical course of disease^62^.

## Conclusion

In the present study, we assessed the immunogenicity of ten non-covalently cell surface-bound proteins of *S. aureus*, using plasma samples from patients with EB and healthy volunteers. Surface-exposed and secreted proteins of *S. aureus* have previously been studied as potential vaccine targets^9,10^. However, while most studies focused on covalently cell wall-bound proteins, the less-studied non-covalently cell wall-bound proteins could also be promising vaccine targets^16^. Therefore, we applied a combined bioinformatics and biochemical approach to select ten non-covalently cell wall-bound proteins of *S. aureus* for further analyses. These included Eap, Efb, EMP, IsaA, LukG, LukH, SA0710, Sle1 and SsaA2, as well as two separated domains of Atl. These potential antigens were expressed in *L. lactis*, purified and tested for antigenicity using human plasma samples. Remarkably, our present results show that the high exposure of EB patients to *S. aureus* was mirrored by significantly elevated IgG levels against all tested non-covalently cell wall-bound antigens. This is a novel finding, which suggests that these antigens on the cell surface of *S. aureus* are prime targets for the human immune system.

## Electronic supplementary material

Supplementary Dataset
